# Editorial: Pre-workout nutrition

**DOI:** 10.3389/fspor.2023.1257740

**Published:** 2023-07-21

**Authors:** Chad M. Kerksick, Jamie N. Pugh

**Affiliations:** ^1^ Exercise and Performance Nutrition Laboratory, College of Science, Technology, and Health, Lindenwood University, St. Charles, MO, United States; ^2^ Research Institute for Sport and Exercise Sciences, Liverpool John Moores University, Liverpool, United Kingdom

**Keywords:** timing, peri-nutrition, post-exercise, pre-exercise, performance, training adaptations

**Editorial on the Research Topic** Pre-workout nutrition

**Figure 1:**
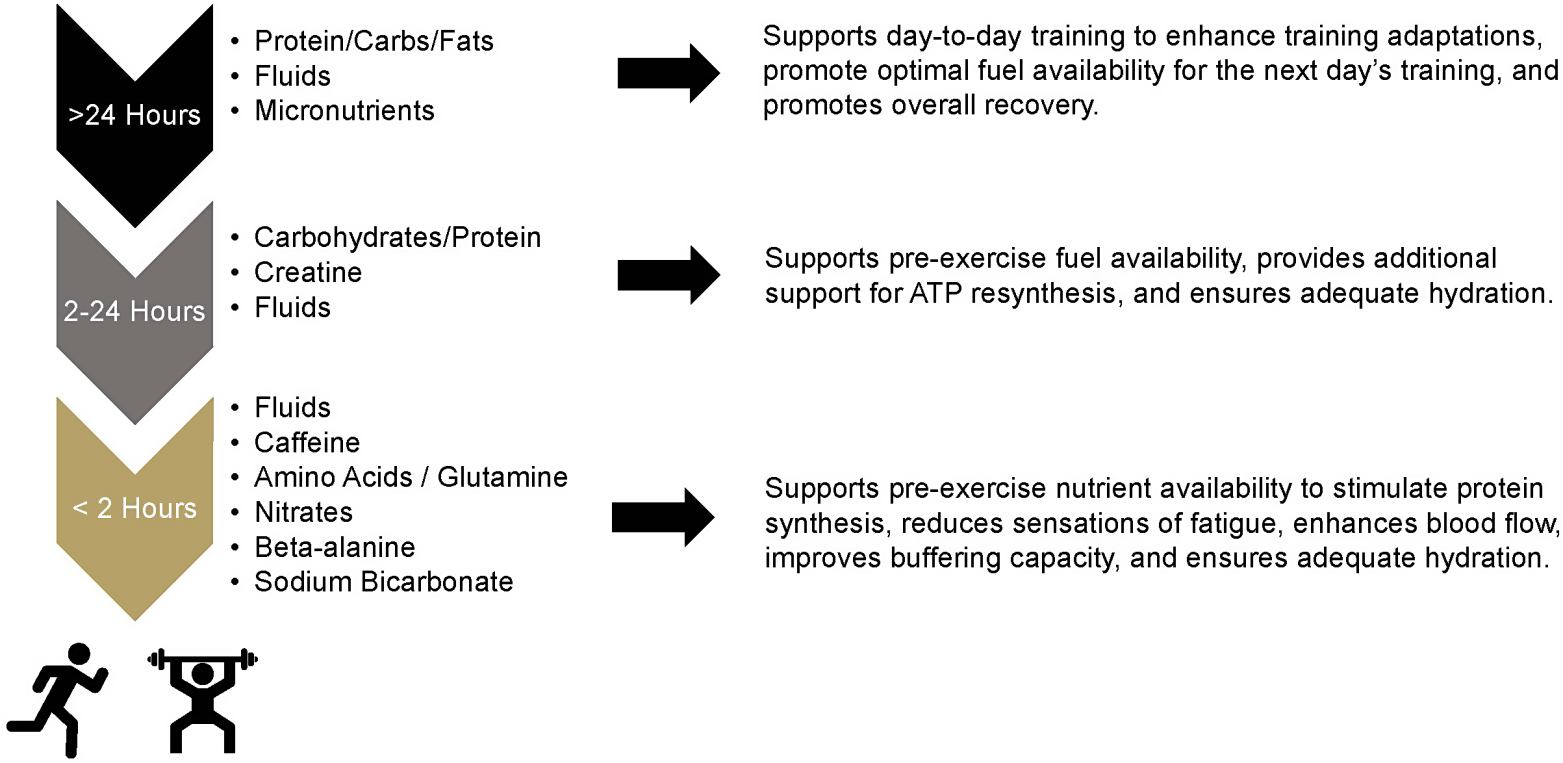
Nutritional ingredients purported to impact acute exercise responses or exercise training adaptations.

For decades, athletes have manipulated what foods they have consumed to maximize their performance and the adaptations their bodies make to the physical training they complete. The roots of nutrient timing date back to the original Scandinavian research of Bergstrom that popularized percutaneous collection of skeletal muscle tissue and made some of the first inferences to the connection between glycogen levels and carbohydrate intake (1). This work initially led athletes to focus on consuming nutrients before and during workouts (2, 3), which led to the first nutrient timing strategy commonly known as glycogen supercompensation or “carbohydrate loading”. Research in the early 1990s then provided some of the first experimental evidence which demonstrated that when nutrients are consumed may impact recovery potential and subsequent performance (4, 5). The popularity of nutrient timing exploded when research by the late Kevin Tipton first suggested that pre-exercise feeding of essential amino acids with carbohydrate may promote higher rates of muscle protein synthesis than when those nutrients were ingested after exercise (6). The flames of this excitement were further stoked by the initial work by Cribb et al. (7) who reported that consuming nutrients in close proximity to a workout was responsible for greater increases in strength and accretion of fat-free tissues. Since that time, research in this area has been consistent with multiple review articles that have summarized the literature base (8–11). While the majority of the work has focused on outcomes surrounding macronutrient administration, recent investigations have started to explore the impact of various micronutrients (8), caffeine (12), and amino acids such as creatine (13) and, most recently, glutamine (14). Figure 1 has been developed to highlight the different ingredients that have been investigated or purported for their ability to impact physical performance or adaptations to physical training when ingested prior to competition or a training session.

The aim of this Research Topic was to provide a direct publication avenue for articles that highlighted relevant topics on nutritional support for athletes including diet composition, supplements, and meal timing surrounding training and sport. As the literature base of nutrient timing has evolved, more research is needed to help scientists, athletes, and practitioners understand how and when nutrient timing should be considered. This Research Topic includes seven original articles that all explored the impact of ingesting different ingredients prior to some form of exercise. Supplementation with ATP has previously been shown to positively impact health and exercise performance, but the optimal dose is unknown. de Moura et al. demonstrated that a 400-mg dose of ATP prior to resistance training exercise was needed to improve performance while lower doses positively impact perceived exertion. Benjamin et al. evaluated the safety of consuming a dose of *Citrus Aurantium* prior to submaximal aerobic exercise and concluded that a 600 mg dose of a 30% extract of p-synephrine was well tolerated and exhibited no unfavorable cardiac and hemodynamic outcomes. An animal study by Che et al. investigated the impact of oral administration of pyruvate on mitigating the onset and accumulation of metabolic acidosis after high-intensity interval exercise. They concluded that a seven-day regimen of oral pyruvate (616 mg/kg/day, HED: 99.4 mg/kg) prior to exercise attenuates the metabolic acidosis induced by high-intensity interval exercise. Three studies in our Research Topic explored the impact of creatine prior to exercise. Negro et al. supplemented 18 adult males with either a placebo, creatine citrate, or a multi-ingredient combination of nutrients containing creatine and found that differences in the time to perform an established task and various EMG parameters were exhibited between the combination of nutrients and placebo. No differences, however, were found between creatine citrate and the other supplementation conditions. Dinan et al. reported that the timing of creatine ingestion (pre- vs. post-workout consumption) in conjunction with a daily dose of protein and carbohydrate was not responsible for any further changes in strength or body composition after 8 weeks of supplementation in 34 healthy resistance-trained male and female collegiate athletes. Candow et al. reviewed the literature on creatine timing and concluded that the current literature is inconclusive on whether or not creatine ingestion or after a workout offers a strategic advantage and put forth a call for researchers to further investigate this topic. Finally, Ratliff et al. used a randomized, crossover design with 15 healthy females to examine the impact of pre-exercise ingestion of carbohydrate or two different sources of protein on energy expenditure and substrate oxidation throughout and after a 60-minute bout of moderate intensity treadmill exercise. The authors concluded that carbohydrate ingestion increased carbohydrate oxidation greater than no nutrient ingestion while no changes were observed for carbohydrate or fat oxidation when either source of protein was consumed prior to each exercise bout. Alternatively, both protein sources triggered increased rates of energy expenditure when compared to carbohydrate ingestion. In summary, the articles submitted to this Research Topic contribute to our understanding of how pre-exercise consumption of different nutrients can impact exercise performance and how we can expect our body to response to regular exercise training.
